# The Chinese Way

**Published:** 1890-02

**Authors:** 


### How Would You Like the Chinese Way?

A Chinese correspondent says: The more I study Americans, the more I am convinced that they are mentally diseased. Instead of doing every thing in a common-sense manner, they try all they can to do it in the very opposite way. At home, for example, you and the other members of your mutual Health Association pay Dr. Wun Lung and his assistants a liberal salary to keep you all well, and pay nothing when you are sick. On this account, he and his young men work very assiduously in regularly calling and examining every member of the union, and all of you enjoy comparative immunity from illness. Here, in New York, a physician is paid for by the amount of your sickness, and the less able you are to earn money, the larger and more onerous is his bill. As a result, many doctors, I am told, yield to temptation and keep their customers sick. The consequence is that those who have the largest number of sick and dying are the richest, most esteemed and influential, while in China they would be ostracised and not allowed to practice.—*Med. Brief.*

